# West Nile Virus Seroprevalence in a Selected Donkey Population of Namibia

**DOI:** 10.3389/fvets.2021.681354

**Published:** 2021-06-18

**Authors:** Umberto Molini, Giovanni Franzo, Hannah Nel, Siegfried Khaiseb, Charles Ntahonshikira, Bernard Chiwome, Ian Baines, Oscar Madzingira, Federica Monaco, Giovanni Savini, Nicola D'Alterio

**Affiliations:** ^1^Faculty of Agriculture and Natural Resources, School of Veterinary Medicine, University of Namibia, Neudamm Campus, Windhoek, Namibia; ^2^Central Veterinary Laboratory (CVL), Windhoek, Namibia; ^3^Department of Animal Medicine, Production, and Health, University of Padova, Padova, Italy; ^4^Istituto Zooprofilattico Sperimentale dell'Abruzzo e del Molise “G. Caporale”, Teramo, Italy

**Keywords:** west nile virus, usutu virus, Namibia, antibody, donkeys, cELISA test

## Abstract

West Nile Virus (WNV) is a mosquito-borne virus enzootically maintained in birds. However, it can incidentally infect other species, leading to sometimes severe clinical consequences like in horses and especially human beings. Despite the topic relevance, the presence and distribution of WNV are currently unknown in Namibia. Several countries implement surveillance systems based on virus detection in birds, mosquitoes, and vertebrate species including horses. The present study aimed to fill this knowledge gap by serologically evaluating WNV exposure in Namibian donkeys, whose population is remarkably bigger than the horse one. Forty-seven out of 260 sampled animals showed neutralizing antibodies against WNV (18.07% [95% CI = 13.59–23.30%]), demonstrating its circulation in all country territory, although, with apparent regional differences. On the contrary, no association with animal age or sex could be identified. The present study demonstrates the widespread presence of WNV in Namibia as well as the practical utility and effectiveness of donkeys as sentinels for infection surveillance. Due to clinical relevance, vaccination campaigns should be considered for horses of high economic or genetic value. Additionally, the burden of WNV infection on human health should be carefully evaluated.

## Introduction

West Nile Virus (WNV) is a mosquito-borne virus of the genus *Flavivirus* within the family *Flaviviridae*. First discovered in 1937 in the West Nile district, Uganda (1), WNV is maintained in nature in an enzootic cycle involving ornithophilic mosquitoes and birds. Birds act as reservoirs and amplifying host while mosquitoes as vectors (2). Humans and horses are incidental or dead-end hosts. Of all the domestic animals, horses are the most commonly affected (3). However, only ~10% of infected horses develop clinical signs (4) and the mortality can reach 50% (4). The susceptibility of other equine species to WNV infection remains poorly documented. WNV antibodies have been found in donkeys and mules in several African countries (5–8). Moreover, WNV is a well-known zoonotic infection that, despite being often asymptomatic, can also lead to flu-like symptoms and, in a minority of cases, to severe neurological complications and even death (9). WNV is endemic to South Africa and serologic surveys conducted in the past confirmed that WNV is widely distributed in horses, wildlife and non-equine domestic species (10, 11). A recent retrospective study showed the presence of the WNV lineage 2 in South African horses with a prevalence of 7.4% (12). Unfortunately, in southern Africa, laboratory investigation has been performed on a small proportion of cases and the burden of WNV is poorly understood or, at least, data collection is inhomogeneous and often outdated, due in part to the limited availability of diagnostic methods across large areas of the continent (3). Large outbreaks of West Nile fever are occasionally reported in human populations (13–15) but are rare, go undiagnosed or are blamed on other viral causes of febrile disease such as yellow fever or dengue. Particularly, limited information is available for WN virus (WNV) in Namibia. The objective of this study was to determine, for the first time, the seroprevalence of WNV in donkeys in Namibia.

## Materials and Methods

### Ethical Approval

Serum samples from a previous research project conducted in Namibian donkeys and authorized by the Animal Research Ethics Committee of the University of Namibia were used. Therefore, no ethical approval specific to this study was needed.

### Sample Collection

Between October 2018 and July 2019, blood samples were collected from 260 donkeys by professional veterinarians in 13 administrative regions of Namibia (20 samples for each region). Samples were randomly collected from a population of healthy donkeys, non-vaccinated against WNV, aged between 3 and 5 years (102 male and 158 female) that had never been out of these regions and whose clinical history did not report clinical signs compatible with WNV infection.

In Namibia, a veterinary cordon fence separates the northern from the southern macro area (i.e., Zambezi, Kavango East, Kavango West, Oshikoto, Ohangwena, Oshana, Omusati, and Kunene regions are located in the northern part of Namibia while Erongo, Otjozondjupa, Omaheke, Khomas, Hardap, and Karas in the southern part). No samples were collected from the Zambezi region because there aren't donkeys in the region. Serum was separated by centrifugation at 3,000 rpm for 5 min, refrigerated, and sent to the Central Veterinary Laboratory (CVL) in Windhoek for testing.

### Serological Tests

Serum samples were analyzed using a commercial competitive ELISA (cELISA) (ID Screen^®^ West Nile Competition Multi-species, IDvet, Grabels, France) which targets antibodies against one epitope of the Pr-E protein of Flavivirus. WNV ELISA positive samples were confirmed using a virus neutralization test (VNT) as described by di Gennaro et al. (16) which is more specific than the ELISA. The VNT was performed at the OIE Reference Laboratory for West Nile Disease, Istituto Zooprofilattico Sperimentale “G. Caporale,” Teramo, Italy.

### Statistical Analyses

The presence of any association between WNV serology results and animal sex or geographical origin or macro area was tested using the Fisher exact test as implemented in the *gmodels* library (17) of R, while the association with animal age was tested using the non-parametric Mann-Whitney test since the age distribution was proven non-normal in the considered sample through data graphical evaluation and Shapiro-Wilk test. A linear model was fitted to evaluate the relationship between animal age and VN titers. The statistical significance level was set to *P*-value < 0.05.

## Results

### Seroprevalence of WNV

A total of 108 out of 260 (41.54% [95% CI = 35.38–47.79%]) analyzed donkeys tested positive by ELISA, and in 47 out of 260 (18.07% [95% CI = 13.59–23.30%]) the presence of WNV neutralizing antibodies was confirmed by VNT (Table 1). Neutralizing titers of 1:10, 1:20, and >1:20 were found in 23 (48.94%), 14 (29.79%), and 10 (21.28%) animals, respectively. WNV circulation was demonstrated in all investigated regions with a prevalence between 5 and 60%. No significant association was found between WNV serological data and sex (*p* = 0.98) or animal age (*p* = 0.44). Similarly, no association was detected between animal age and WNV neutralizing titers (*p* = 0.29), although, a slightly decreasing trend was observed with increasing animal age. While no differences were observed between the WNV prevalence in the northern and southern part of the country (*p* = 0.15), the geographical origin of animals significantly (*p* < 0.001) influenced the prevalence of infection. Based on the analysis of the standardized residual, Oshikoto demonstrated a significant excess of positive results compared to what expected by chance alone.

**Table 1:** Seroprevalence of WNV in donkeys according to Namibian regions.

**Region**	**c-ELISA**	**VNT WNV**
Kavango east	12/20 (60% [36.05–80.88])	7/20 (35% [15.39–59.22])
Kavango west	9/20 (45% [23.06–68.47])	1/20 (5% [0.13–24.87])
Kunene	9/20 (45% [23.06–68.47])	1/20 (5% [0.13–24.87])
Erongo	1/20 (5% [0.13–24.87])	1/20 (5% [0.13–24.87])
Omaheke	9/20 (45% [23.06–68.47])	1/20 (5% [0.13–24.87])
Omusati	8/20 (40% [19.18–63.95])	6/20 (30% [11.89–54.28])
Hardap	9/20 (45% [23.06–68.47])	5/20 (25% [8.66–49.10])
Karas	2/20 (10% [1.24–31.70])	2/20 (10% [1.24–31.70])
Khomas	12/20 (60% [36.05–80.88])	4/20 (20% [5.73–43.66])
Otjozondjupa	5/20 (25% [8.66–49.10])	2/20 (10% [1.24–31.70])
Oshikoto	17/20 (85% [62.11–96.79])	12/20 (60% [36.05–80.88])
Ohangwena	6/20 (30% [11.89–54.28])	3/20 (15% [3.21–37.89])
Oshana	9/20 (45% [23.06–68.47])	2/20 (10% [1.24–31.70])
**Total**	**108/260 (41.54% [35.48–47.79])**	**47/260 (18.07% [13.59–23.30])**

*95% confidence intervals are reported within squared brakets. ELISA, Enzyme-linked immunosorbent assay; WNV, West Nile Virus, VNT, Virus Neutralization Test*.

## Discussion

Donkeys have been suggested as useful sentinel animals for WNVserosurveilance as part of epidemiological monitoring efforts (18). One hundred and eight out of 260 samples (41.54% [95% CI = 35.38–47.79%]) tested positive using a competitive ELISA test, although, only 47 (18.07% [95% CI = 13.59–23.30%]) were confirmed by the reference WNV VNT. The large number of samples positive by cELISA and negative by WNV VNT (61/260) may have been due to the limited specificity (Sp) of the cELISA, although, the Sp of the used cELISA has been reported up 95% (19). Cross-reaction with other antigenically-related flaviviruses could also explain the discrepancies in the results of these assays (20).

Positive samples were found in all of the tested regions, suggesting that WNV is present throughout Namibia, although, the seroprevalence found in this study was remarkably lower than previously reported from donkeys and horses in several other African countries (6–8). However, low seroprevalence levels were previously reported in other African countries also (5). A significant difference was found in the number of WNV cases detected in each region. The regions with evident different case numbers were the Kavango East and Oshikoto Regions. Particularly, the Oshikoto Region had a significantly higher number of cases than the other regions of Namibia, while the Kavango West, Erongo, Omaheke and Kunene Regions had lower numbers of positive WNV cases. However, the limited number of donkeys that was possible to sample from each region requires caution when dealing with local WNV prevalence differences. Further, studies based on larger sample size for each region will be necessary to increase the power and precision of the study and to confirm current findings, as well as to investigate the role of additional risk factors.

Differently from African horse sickness, no overall differences were observed between the northern and southern part of the country (21). Potentially, the different vector ecology and distribution could explain the observed difference. The heterogenicity in exposure among the populations of donkeys in the 13 Namibian regions may result from different climate and rainfall intensity between areas, which influence the vector abundance. Unfortunately, the lack of adequate knowledge of WNV main vectors in Namibia prevents any definitive conclusion and further studies should be dedicated to this topic. There was no statistically significant relationship between the number of WNV cases and age or sex. The low viral titer typically detected suggests that viral infection was not recent. Accordingly, although, a slightly decreasing trend was observed with increasing animal age. Studies based on viral detection rather than seroprevalence could be of help in the investigation of infection timing and the potential occurrence and severity of clinical signs. Although, cases of West Nile fever were never recorded in equids around Namibia, the results of this study provided enough evidence about the presence and viral circulation all around the country, testifying the practical usefulness end efficacy of routinary serological monitoring of donkeys as sentinels for WNV presence. Surveillance systems based on virus detection in birds, mosquitoes, horses and poultry are used in several countries to estimate the public health risk for flaviviruses such as WNV (21). A livestock census conducted in Namibia in 2015 showed a population of 27.151 horses and 148.859 donkeys (22). Due to the significantly larger population of donkeys in Namibia when compared to that of horses, donkeys can be a valuable resource for disease surveillance and as sentinels for a national integrated surveillance system (“One Health”) to prevent emerging flavivirus infection. Finally, the circulation of WNV in the region might suggest that horses, especially those with high genetic and/or economic value, should be prudentially vaccinated throughout Namibia against WNV.

## Data Availability Statement

The original contributions presented in the study are included in the article/supplementary material, further inquiries can be directed to the corresponding author/s.

## Author Contributions

UM designed, coordinated, carried out the survey, and wrote the manuscript. HN and CN carried out the survey. GF, GS, and SK analyzed the data. BC, IB, and OM collected the samples. GF and GS wrote and edited the manuscript. FM designed the study. ND'A supervised the study. The final manuscript has been read and developed in consultation with all authors. All authors have read and approved the final manuscript.

## Conflict of Interest

The authors declare that the research was conducted in the absence of any commercial or financial relationships that could be construed as a potential conflict of interest.
